# An in-home intervention of parent-implemented strategies to increase child vegetable intake: results from a non-randomized cluster-allocated community trial

**DOI:** 10.1186/s12889-019-7079-4

**Published:** 2019-07-04

**Authors:** Francine M. Overcash, Zata Vickers, Allison E. Ritter, Traci Mann, Elton Mykerezi, Joseph Redden, Aaron K. Rendahl, Cynthia Davey, Marla Reicks

**Affiliations:** 1 Department of Food Science and Nutrition, University of Minnesota, 1334 Eckles Ave, St. Paul, MN 55108 USA; 2 Department of Psychology, University of Minnesota, N218 Elliott Hall, 75 E. River Road, Minneapolis, 55455 USA; 3 Department of Applied Economics, University of Minnesota, 1994 Buford Ave, St. Paul, MN 55108-6040 USA; 4 Carlson School of Management, University of Minnesota, 321 19th Ave S, Ste. 3-161, Minneapolis, MN 55455 USA; 5 College of Veterinary Medicine, Statistics and Informatics, University of Minnesota, 295L ASVM Building, 1988 Fitch Avenue, Saint Paul, MN 55108 USA; 6 Clinical and Translational Science Institute, University of Minnesota, 717 Delaware Street SE, Second Floor, Minneapolis, MN 55414 USA

**Keywords:** Vegetable intake, Child, Intervention, Behavioral economics

## Abstract

**Background:**

Less than 2% of children in the U.S., ages 9–13, meet the minimum dietary recommendations for vegetable intake. The home setting provides potential opportunities to promote dietary behavior change among children, yet limited trials exist with child vegetable intake as a primary outcome. Strategies to increase vegetable intake grounded in behavioral economics are no/low cost and may be easily implemented in the home by parents.

**Methods:**

This non-randomized, controlled study tested whether an intervention of parent-led strategies informed by behavioral economics and implemented within a series of 6 weekly parent-child vegetable cooking skills classes, improved dietary outcomes of a diverse sample of low-income children (ages 9–12) more than the vegetable cooking skills classes alone. The primary outcomes were total vegetable intake, dietary quality (HEI scores), total energy intake, vegetable liking, variety of vegetables tried, child BMI-z score, and home availability of vegetables. Outcome measures were collected at baseline, immediate post-treatment, 6 and 12 months follow-up. Mixed model regression analyses with fixed independent effects (treatment condition, time point and treatment condition x time interaction) were used to compare outcomes between treatment conditions.

**Results:**

A total of 103 parent/child pairs (intervention = 49, control = 54) were enrolled and 91 (intervention = 44, control = 47) completed the weekly cooking skills program. The intervention did not improve child total vegetable intake. Intervention children increased dark green vegetable intake from immediate post-treatment to 12 months. The number of vegetables children tried increased and mean vegetable liking decreased over time for both control and intervention children.

**Conclusions:**

Findings from this study suggest that the strategies and the manner in which they were implemented may not be effective in low-income populations. The burden of implementing a number of strategies with potentially higher food costs may have constrained the ability of families in the current study to use the strategies as intended.

**Trial registration:**

This trial has been retrospectively registered at : #NCT03641521 on August 21, 2018.

**Electronic supplementary material:**

The online version of this article (10.1186/s12889-019-7079-4) contains supplementary material, which is available to authorized users.

## Background

Despite evidence that vegetables promote diet quality [1], only 2% of early adolescent children (ages 9–13), meet the minimum dietary recommendations for vegetable intake [2]. Early adolescence is a period of development when children begin to establish independence [3], thus reflecting a window of opportunity to guide their food choices toward healthier options. Studies on longitudinal tracking of food preferences and eating patterns from childhood and adolescence [4] into adulthood [5] support interventions to increase vegetable intake before adolescence.

The home is a setting rich with opportunities to promote dietary behavior change among children, yet limited trials exist with child vegetable intake as a primary outcome. Almost 70% of daily calories are consumed at home [6]. Meals prepared from scratch at home have higher diet quality and result in greater vegetable intake compared to meals consumed away from home [7, 8]. The few interventions set in the home have produced mixed results [9]. For example, Remington and colleagues tested if parent-administered repeated taste exposures paired with reward incentive in the home would increase intake and liking of vegetables among young children compared to a no-treatment control group [10]. A positive intervention effect was observed for both liking and intake of the target vegetable, which was maintained at 3-months follow-up. Whereas Fulkerson et al. [11] found that a family-focused cooking skills and taste exposure intervention (HOME Plus) implemented by parents in their homes did not increase children’s overall vegetable intake. Home implementation of a video and taste exposure program (*Food Dudes*) was also not effective in increasing child vegetable intake compared to a control group, [12] despite the same intervention procedure increasing vegetable intake among children during lunch in a school cafeteria setting [13]. A recent systematic review concluded that inconsistent findings support the need to better understand how to successfully increase vegetable intake among children using parent-led in-home intervention strategies [14].

Behavioral economics is a relatively new field that, when applied to food choice, has the potential to produce widespread nutritional benefit [15]. It utilizes principles of libertarian paternalism (the ability to affect behavior without limiting choice), ‘nudges’ (small actions that influence choice), and choice architecture (organizing the context in which people make decisions) [16, 17]. Behavioral economics posits that slight modifications to the social and physical environments may shift behaviors toward an optimal state without limiting choice [16–18].

The majority of interventions informed by behavioral economics to increase vegetable consumption have been conducted in the school setting, with few trials testing similar strategies at home. Successful strategies in schools involved serving vegetables first and in isolation [19, 20], improving the presentation of vegetables (e.g., colorful pictures in lunch trays) [21], increasing the convenience and visibility of vegetables [22, 23] increasing the variety of vegetables offered [24] and increasing the portion size of the vegetable served [25]. Cravener et al. [26] conducted a small pilot control-intervention study (*n* = 24) based in the home to increase vegetable intake of young children identified as low-vegetable consumers. They tested the effectiveness of pairing the strategies of making vegetables the default option and making them the more attractive snack (i.e., cartoon character packaging). The paired strategies increased vegetable intake by 1 vegetable serving/day. Leak et al. [27] tested for differences in vegetable intake between a number of parent-led behavioral strategies among parents of 9–12 year old low-income children who implemented a different strategy weekly for 6 weeks at home. *Serving two vegetables* with the dinner meal resulted in significantly greater vegetable intake relative to two other strategies: *pairing vegetables with other foods the child likes* (0.43 more cups; *p* = 0.01) and e*ating dinner together with an adult(s)* (0.39 more cups; *p* = 0.04). de Wild and colleagues found that offering a choice of vegetables (i.e., 2 vegetables versus 1 vegetable) for the meal at home did not predict vegetable intake for a group of 70 children aged 2–5 years [28]. A recent review of experimental trials to improve children’s eating behavior concluded the low-cost of behavioral economics strategies supports the need for more of these types of interventions [29]. Given the low economic burden, positive results from school-based studies and mixed results from home-based studies, further studies in the home, especially among older children, are warranted.

Factors that affect vegetable intake of children include individual, environmental, and behavioral influences. For example, children’s individual liking of vegetables affects motivation and intention (behavior), which affects intake, especially in youth who are known to eat what they like and avoid what they dislike [30–32]. Repeated exposure has been effective in improving intake of vegetables among infant and pre-school children [32–34]. Far fewer trials testing the efficacy of repeated exposure on increasing vegetable intake for older children exist, although one trial based on repeated exposure as a strategy showed improved liking of previously disliked vegetables among school-aged children [35]. Availability of vegetables is a well-established environmental predictor of child and adolescent dietary choices and consumption [36], which are influenced by parental modeling (behavior) [36, 37]. Interventions that address factors associated with vegetable intake such as liking and availability may be effective in improving and sustaining behavioral change.

The primary objective of this study was to test whether an intervention of parent-led strategies informed by behavioral economics and implemented within a series of 6 weekly parent-child vegetable cooking skills classes, improved dietary outcomes of a diverse sample of low-income children (ages 9–12) more than the vegetable cooking skills classes alone.

This controlled intervention trial also tested the intervention’s effectiveness for child liking of vegetables, variety of vegetables tried, home availability of vegetables, child dietary quality, child total energy intake, and child Body Mass Index (BMI).

## Methods

### Overview of study design

**Fig. 1:**
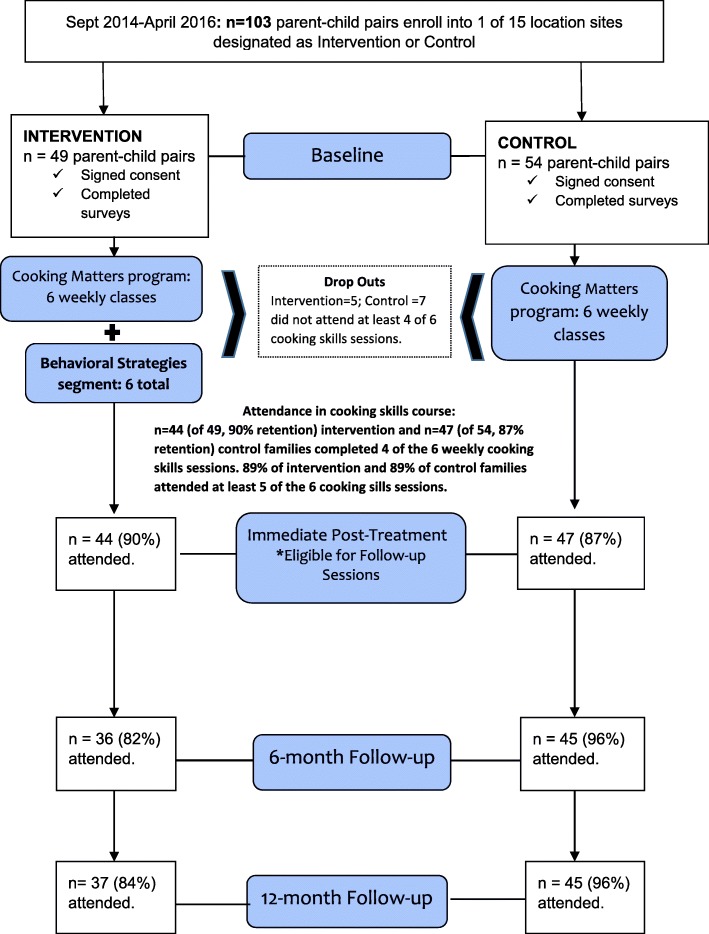
Study Flow Chart

This was a cluster-allocated controlled intervention trial set in low-income communities throughout the Minneapolis/St. Paul metropolitan area. Parent/child (9–12 years) pairs were recruited to be balanced among intervention and control conditions at locations sites (Fig. 1). All outcome measurements were collected at 4 time points: baseline, immediate post-treatment, 6-month follow-up, and 12-month follow-up.

Share our Strength’s® Cooking Matters for Families cooking skills program (6 total classes) (http://www.cookingmatters.org), was enhanced to provide greater emphasis on vegetable preparation, procurement, and intake. Both intervention and control families participated in the enhanced, vegetable-focused, Cooking Matters for Families cooking skills program. The only difference between treatment conditions was that parents in the intervention condition learned a new behavioral economics-informed strategy in the each of the 6 classes, while these strategies were not introduced to parents in the control condition. The parents were asked to practice the new strategy in their homes each week. Each behavioral strategy was matched to one of the vegetable-focused, cooking skills classes and recipes that would best promote or complement the strategy. Strategies were tested for feasibility in a pilot study [27]. The vegetable-focused, cooking skills classes were intended to enhance the ability of parents in the intervention condition to practice the behavioral strategies at home and decrease any perceived barriers. Participation of the control families in the vegetable-focused, cooking skills classes but without introduction of the behavioral strategies represented an appropriate control condition allowing for the strategies to be tested under optimum conditions.

Cooking Matters has been shown to improve cooking confidence, food resource management, and decrease healthy cooking barriers in adults [38]. Social Cognitive Theory [35] served as the basis for the Cooking Matters program addressing behavioral outcomes such as child vegetable intake, personal factors such as vegetable liking and environmental factors such as home vegetable availability (Additional file 1: Figure S1).

### Participants

Parent-child pairs were recruited primarily through flyer/email campaigns at location sites serving low-income families including subsidized housing, schools, churches, and community centers. A sample size calculation estimated that 100 parent/child pairs, equally distributed between the intervention condition and the control condition were required. This number would allow for detection of an effect size (vegetable consumption in servings) of 0.57 standard deviations with 80% power accounting for a 20% drop out rate. Eligibility criteria included: 1) participant child must be 9–12 years old; 2) parent must be the main food preparer for the household, 3) the family must qualify for some form of public assistance, 4) have a phone, 5) must not have participated in a previous Cooking Matters for Families in the past 3 years; and 6) be able to read, speak, and understand English (or Spanish for Spanish-only classes). To enhance study retention, parents and children were paid a total of $200 and $60, respectively, spread across the 4 time points when outcome measurements were collected. The University of Minnesota Institutional Review Board approved the study (Study number: 1111S06501). The study adheres to CONSORT guidelines.

### Location sites

From September 2014 to June 2016, the study was held at 15 location sites (8 intervention, 7 control) throughout the Minneapolis-St. Paul Metro area (Table 1). Location sites were identified using the University of Minnesota Extension Cooking Matters network. Although randomization of participant families at each location site was intended, low numbers of enrolled families were not sufficient to hold both a control and intervention condition. As a result, location sites were allocated to either the control or intervention treatment condition. In order to keep each treatment condition balanced, the number of enrolled families in each condition to date determined whether a control or intervention condition would be held at future location sites. Intervention and control treatment conditions were held at location sites with similar service missions and participant demographic characteristics (Table 1). Enrollment across location sites ranged from 4 to 16 parent-child pairs.

**Table 1:** Description of Location Sites

Description	Program Date	Follow-up Dates	Number of parent/child pairs^a^
Subsidized public housing with 592 rental units, St. Paul	Fall 2014	6-mo: Mar 2015 12-mo: Aug 2015	C = 6 enrolled; 1 dropped out
Subsidized public housing with 302 rental units, St. Paul	Fall 2014	6-mo: Mar 2015 12-mo: Aug 2015 6 mo: Aug 2016	I = 6 enrolled; 6 completed
	Winter 2016	12 mo: Feb 2017	C = 10 enrolled, 8 completed
Private Catholic Middle School; 95% Hispanic; sessions conducted in Spanish, South Minneapolis	Winter 2015	6-mo: May 2015 12-mo: Jan 2016	I = 7 enrolled; 6 completed C = 6 enrolled; 6 completed
Subsidized public housing; single parent households with children, St. Paul	Spring 2015	6-mo: Sept 2015 12-mo: May 2016	C = 6 enrolled; 5 completed
Subsidized public housing; families with children, homeless, recovering from chemical dependency, St. Paul	Spring 2015	6-mo: Sept 2015 12-mo: May 2016	I = 7 enrolled; 7 completed
Catholic Church, primarily serving Hispanic population; Sessions conducted in Spanish, Richfield	Summer 2015	6-mo: Dec 2015 12-mo: June 2016	C = 16 enrolled; 15 completed
Transitional housing; families who have been homeless; 20 units, White Bear Lake	Summer 2015	6-mo: Jan 2016 12-mo: July 2016 6-mo: Sept 2016	I = 7 enrolled; 6 completed
	Spring 2016	12-mo: Apr 2017	C = 5 enrolled; 3 completed
Community agency serving Latino families, Minneapolis	Fall 2015	6-mo: Mar 2016 12-mo: Oct 2016	I = 8 enrolled; 7 completed
Affordable apartment community; on-site YMCA programs; 168 units, Maplewood	Fall 2015	6-mo: Mar 2016 12-mo: Nov 2016	C = 5 enrolled; 5 completed
Affordable apartment community; on-site YMCA programs; Little Canada	Winter 2016	6-mo: Aug 2016 12-mo: Jan 2017 6 mo: Nov 2016	I = 6 enrolled; 5 completed
	Spring 2016	12 mo: May 2017	I = 5 enrolled; 4 completed
Section 8 Housing complex; on-site YMCA programs, Minnetonka	Spring 2016	6 mo: Oct 2016 12 mo: Apr 2017	I = 4 enrolled; 3 completed

^a^I = Intervention, C = Control

Four location sites (2 intervention, 2 control) were Spanish-only, led by a bilingual nutrition educator. Outcome measurement tools were translated into Spanish and back-translated into English prior to use, except for the Cooking Matters for Families instrument which was available in Spanish.

### Procedure

The format of each 2 h Cooking Matters class (6 total), delivered to both control and intervention conditions, consisted of 1) a professional chef demonstrating a recipe, 2) parent/child pairs preparing the recipe under the guidance of both the chef and a nutrition educator, 3) a trained nutrition educator delivering a nutrition education lesson, and 4) participants eating a family-style meal prepared by the participants and chef during the class. Families took home a bag of groceries that included all the ingredients used during the class to prepare the meal at home.

Intervention parents participated in an additional 20–25-min segment led by the nutrition educator during which the week’s behavioral strategy was introduced. The following six behavioral strategies were introduced (one each week) as a segment within each cooking skills class: 1) have your child help prepare vegetables for meals (*Child Help*), 2) use a plate that shows the amount of vegetables to include for a meal (*My Plat*e), 3) make vegetables visible and accessible by removing other foods from the dining area during the meal and leaving the vegetables (*Make Avail/Visible*), 4) serve at least 2 vegetables with the meal (*Serve 2*), 5) serve vegetables before the meal (*Serve First*), and 6) use a bigger spoon to serve the vegetables (*Big Spoon*). Behavioral strategies were matched to one of the vegetable-focused, cooking skills classes and recipes that would best promote use of the strategy at home. For example, the strategy *Serve 2* was introduced in the class entitled “The Power of Planning”. In this class, parents discussed how meal planning in advance would make serving 2 vegetables for dinner easier to implement. The class entitled “Cooking Side-by-Side” was matched to the behavioral strategy of having the child help prepare the vegetables for the meal.

Preceding study launch, nutrition educators attended a series of trainings led by the study team. Nutrition educators followed the same scripted format for each intervention segment: 1) introduce the new strategy, 2) provide examples for implementation, 3) troubleshoot problems with implementation, and 4) assist with plans to use the strategy. During the intervention segment in the week following the introduction of the new strategy, the nutrition educator asked how the strategy was implemented, what barriers and facilitators affected use of the strategy, and whether the strategy improved child vegetable intake. Some strategies required additional supplies such as acrylic *My Plates* and a *Big Spoon* (1/2 cup/240 ml) that were given in the take-home grocery bags for the families.

### Outcome measures

#### Dietary intake

Child vegetable intake, child total energy intake, and dietary quality (i.e., Healthy Eating Index 2010 scores), were assessed using 24-h dietary recalls collected via Nutrition Data System for Research (NDSR) software (versions 2014–2016), a validated dietary analysis program supported by the Nutrition Coordinating Center (NCC), at the University of Minnesota, Minneapolis, Minnesota. Research staff trained and certified by NCC staff collected three 24-h dietary recalls in-person during and over the phone immediately following of the 4 outcome measurement time points. Research staff prioritized collecting one weekend-day and 2 non-consecutive weekdays, a combination that has been reported as “ideal” in assessing usual intake [39]. To increase accuracy of the recalls, staff used visual aids (e.g., cups, bowls, measurement tools, and models of vegetables) and the NDSR Food Amounts Booklet when conducting the recalls in person, and asked for parental assistance if necessary. Individual daily recalls were excluded (*n* = 29) if they were deemed biologically implausible (i.e., < 500 kcal/day or if total vegetable intake exceeded 7 cups). Total vegetable intake, total non-fried vegetable intake, and total energy intake were generated using NDSR (version 2016) output reports. Vegetable and total energy intake data were averaged across days for each time point. Healthy Eating Index (HEI) 2010 scores [40] were calculated to assess dietary quality. After formatting the dietary recall data for analysis per NCC guidelines, total HEI and HEI vegetable component scores were calculated using a SAS® program (version 9.4) (SAS Institute Inc. Cary, NC 2014), created by National Institutes of Health-NCI, Division of Cancer Control & Population Studies [41].

#### Child liking and variety

Children rated their liking of 37 different vegetables on an iPad® survey (QuickTapSurvey®). The question “Have you ever tried (name of vegetable)” was first asked for each vegetable. If the child answered ‘yes,’ the next screen asked the child to rate their liking of the vegetable by sliding a finger across a 10-point labeled hedonic scale (1-“Hate it” to 5- “It’s okay” to 10-“Love it”). If they answered ‘no,’ the next screen asked about “ever trying” the next vegetable. A handout with colored pictures of all the vegetables presented in the same order as in the survey was provided as a visual aid. An aggregate vegetable liking score representing mean liking rating for all vegetables for all children by treatment condition was calculated. Variety was calculated by summing the number of vegetables for which the child answered “yes” to the “ever tried” question.

#### Home vegetable availability

Parents were asked to complete a validated Home Food Inventory, developed by Fulkerson and colleagues [42], adapted for the current study to self-report the availability of 35 vegetables currently in their home. The home food inventory included a 1-page cover sheet with instructions to inspect all areas of the home where vegetables were stored (refrigerator, freezer, pantries, and cupboards). Vegetables were listed in separate rows in a checklist type format with “yes/no” response options to check for 3 columns per row: fresh, can/jar, frozen. To improve accuracy of the self-reported inventory, research staff reviewed the instructions with parents and asked participants to complete an example form during the baseline session.

#### Child BMI-z score

Child height and weight were measured by trained research staff using a stadiometer (model: Seca 202, Hanover, MD) and a digital weight scale (model: Tanita BWB-800P Digital Medical Scale, Arlington Heights, IL, USA). Three measures of both height (to the nearest 0.1 cm) and weight (to the nearest 0.1 kg) were collected and averaged at each of the 4 time points. For measurement accuracy, children were asked to remove their shoes and change into a t-shirt and athletic shorts. BMI-z scores were generated using a SAS program created by the Centers for Disease Control and Prevention [43].

### Statistical analysis

Baseline comparisons for demographic and other variables between intervention and control conditions were performed using independent two-sample t-tests for normally distributed continuous variables, nonparametric tests for non-normally distributed continuous variables, and Chi-square and Fisher’s exact tests for categorical variables. Means and standard deviations were calculated for all outcomes at each of the 4 time points. Comparisons of outcomes between children in the intervention versus children in the control condition used mixed model regression analyses with fixed independent effects including condition (intervention vs. control), time point (baseline, immediate post-treatment, 6-month and 12-month), and treatment *condition x time point* interaction. A random effect for location site was included in the models to account for the additional component of variance associated with the cluster allocation of treatment where observations from children at the same location site may be correlated. A repeated statement was used in the models to account for within-subject correlation of repeated measures (i.e., the 4 outcome measurement time points)**.** The repeated measures are nested within subjects (i.e., children), subjects are nested within location sites, and locations sites are nested within treatment conditions. Mixed model regression appropriately accounts for correlation among repeated measures on the same subject, correlation within clusters, and addresses missing data through valid estimates for all available data [44]. By necessity, locations sites were assigned to either intervention or control but there is not an interest in intervention effect at the location site level. The models account for clustering of location sites within treatment condition. Estimate statements were used to compare adjusted least square means (LSM) by treatment condition to determine between-treatment condition differences at each of the 4 time points. Estimate statements were also used to compare adjusted least square means at each time point to determine within-treatment condition changes over time. To compare change-over-time in outcomes between treatment conditions, change from baseline to each post treatment time point (immediate, 6-months, and 12-months) was calculated for each outcome measure. Adjusted mixed models with a random effect for location site (as described above) were also used for the change-over-time outcomes. As there was interest in each of the analysis outcomes, many of which were correlated, no adjustment for multiple comparisons was made using Bonferroni or other adjustment method. The results of all pairwise comparisons were reported so that the reader can assess the effect of multiple hypothesis testing. Final models for vegetable intake, HEI-2010 scores, child liking of vegetables and child variety of vegetables tried were adjusted for child gender and child age. Other covariates were considered if they had been known to be associated with the outcome. Final model co-variates for each outcome were determined using Bayesian information criterion (BIC) criteria for best model fit. Statistical significance was set at *p* < 0.05. Data management and analyses were conducted using SAS software version 9.4 [45].

## Results

### Attendance

A total of 103 parent/child pairs (intervention = 49, control = 54) were enrolled in the study (Fig. 1). Eighty-nine percent of dyads from both the intervention and control conditions attended at least 5 of the 6 cooking skills classes. Five intervention parent-child pairs and 7 control parent-child pairs did not complete at least 4 cooking skills classes and were not asked to attend follow up outcome measurement sessions. Families reported missing the cooking skills classes because they did not have enough time to participate, obtained a new job that conflicted with weekly classes, or their child preferred not to participate. Of the 44 intervention families invited to attend the follow-up outcome measurement sessions, 36 attended the 6-month follow-up (82% retention rate) and 37 attended the 12- month follow-up (87% retention rate). Of the 47 control families invited to attend the follow-up outcome measurement session, 45 attended both the 6-month follow-up and 12-month follow-up (96% retention rate for both sessions).

### Baseline demographic characteristics

The intervention families did not significantly differ from the control families in any of the baseline demographic characteristics (Table 2).

**Table 2:** Frequencies and Percentages of Baseline Parent, Household & Child Characteristics

Characteristic	Control (*n* = 54)	Intervention (*n* = 49)
	frequency (%)	frequency (%)
Parent sex		
Female	53 (98)	44(89)
Male	1 (2)	5 (10)
Parent age		
18–29	7 (13)	8 (16)
30–39	30 (57)	25 (51)
40–60+	16 (30)	16 (33)
Parent education		
< high school diploma	10(19)	16(34)
High school diploma or GED	23(43)	9(19)
Some college/2-year degree	17(32)	19(40)
4-year college degree	4(7)	3(6)
Parent Race		
White	8(15)	7(14)
Black/African American	23(43)	13(27)
Asian/Pacific Islander/American Indian	0(0)	4(8)
Other	18(33)	21(43)
Mixed Race	5(9)	4(8)
Parent Hispanic Ethnicity	22(41)	19(39)
Household size		
≤ 3	10(19)	8(16)
4–5	25(46)	32(65)
6 or more	19(35)	9(18)
Food Security		
Food Secure	23(43)	15(31)
Low Food Security	16(30)	21(44)
Very Low Food Security	14(26)	12(25)
Child sex		
Female	39(72)	27(55)
Male	15(28)	22(45)
Child age		
9	20(37)	15(31)
10	20(37)	13(27)
11	8(15)	11(22)
12	6(11)	10(20)
Child race		
White	8(15)	7(14)
Black/African American	23(43)	13(27)
Asian/Pacific Islander/American Indian	0(0)	4(8)
Other	18(33)	21(43)
Mixed Race	5(9)	4(8)
Child Hispanic Ethnicity	23(43)	20(41)
Child BMI percentile category		
normal (≥ 5 < 85)	27(50)	26(53)
overweight (≥ 85 < 95)	8(15)	12(25)
obese (≥ 95)	19(35)	11(22)

### Dietary outcome measures

Total vegetable intake as well as intake for all but 1 of the individual vegetables measured (legumes at baseline, *p* = 0.04) did not significantly differ between the intervention and control children at any of the 4 time points (Table 3). An increase was observed for the intervention children’s dark green vegetable intake from immediate post-treatment (LSM = 0.08 servings) to 12-month follow-up (LSM = 0.23) (*p* = 0.05) (data not shown). Control children did not increase their dark green vegetables over the same time period. Based on between treatment condition comparisons of change-over-time measures, control children decreased white potato intake from baseline to 6-months while intervention children increased intake (LSM _control_ = − 1.3, LSM _intervention_ = 0.12, *p* = 0.02) (data not shown).

**Table 3:** Control vs. Intervention - Least Square Means (LSM) of Dietary Intake Outcome Measures at Baseline, Immediate Post-Treatment, 6 Mo Follow-up, and 12 Mo Follow-up

	Baseline			Immediate Post-Treatment			6 Mo Follow-Up			12 Mo Follow-up		
	Control	Intervention		Control	Intervention		Control	Intervention		Control	Intervention	
Outcome Measure	LSM(SE)	LSM(SE)	t-test *p*-value	LSM(SE)	LSM(SE)	t-test p-val	LSM(SE)	LSM(SE)	t-test p-val	LSM(SE)	LSM(SE)	t-test p-value
Total Vegetable Servings ^a,b,^	1.6 (0.2)	1.7(0.2)	0.85	1.7(0.2)	1.5(0.2)	0.53	1.8(0.2)	1.7(0.3)	0.77	1.7(0.2)	1.9(0.1)	0.54
Total Non-Fried Vegetable Servings^a,c^	1.6(0.2)	1.6(0.2)	0.93	1.6(0.2)	1.3(0.2)	0.32	1.7(0.2)	1.6(0.2)	0.82	1.4(0.2)	1.7(0.2)	0.39
Dark Green Vegetable Servings^a,d^	0.1(0.0)	0.1(0.0)	0.91	0.2(0.0)	0.1(0.0)	0.30	0.1(0.0)	0.1(0.1)	0.74	0.1(0.0)	0.2(0.1)	0.32
Deep Yellow Vegetable Servings^a,e^	0.1(0.1)	0.2(0.1)	0.33	0.1(0.0)	0.2(0.0)	0.31	0.1(0.0)	0.2(0.1)	0.06	0.1(0.0)	0.2(0.1)	0.23
Tomato Servings^a, f^	0.5(0.1)	0.4(0.1)	0.27	0.5(0.1)	0.3(0.1)	0.08	0.5(0.1)	0.4(0.1)	0.10	0.4(0.1)	0.4(0.1)	0.95
Legume Servings^a,g^	0.2(0.0)	0.1(0.0)	0.04	0.1(0.1)	0.1(0.1)	0.77	0.2(0.1)	0.1(0.1)	0.14	0.2(0.1)	0.2(0.1)	0.85
White Potato Servings^a,e^	0.2(0.1)	0.2(0.1)	0.83	0.1(0.1)	0.1(0.1)	0.81	0.1(0.1)	0.2(0.1)	0.06	0.2(0.1)	0.1(0.1)	0.61
Total Energy (Kcal)^h^	1588.8 (87.8)	1573.6 (88.7)	0.90	1574.2 (95.5)	1434.3 (94.6)	0.30	1525.0 (97.3)	1577.8 (105.2)	0.7	1428.2 (98.3)	1537.7 (102.8)	0.4
HEI 2010 Total Score^i^	54.8 (2.8)	55.6 (2.7)	0.84	54.5 (2.5)	56.3 (2.4)	0.6	56.8 (2.6)	54.3 (2.7)	0.5	55.8 (2.6)	56.8 (2.6)	0.8
HEI 2010 Total Vegetables Component Score^c^	2.4 (0.2)	2.4 (0.3)	0.84	2.5 (0.3)	2.3(0.3)	0.7	2.6 (0.3)	2.2 (0.3)	0.3	2.6 (0.3)	2.6 (0.3)	1.0
HEI 2010 Greens and Beans Component Score^c^	2.2 (0.3)	1.7 (0.3)	0.35	2.3 (0.4)	1.8(0.4)	0.3	2.5 (0.4)	1.4 (0.4)	0.06	2.2 (0.4)	2.2 (0.4)	1.0

^a^Mean daily values from three 24-h dietary recalls (Nutrition Data System for Research software version 2014, Nutrition Coordinating Center, University of Minnesota, Minneapolis, MN). Vegetable servings are defined per the 2000 Dietary Guidelines for Americans as 1 cup of raw leafy vegetables or ½ cup of other cooked or raw vegetables. Vegetable servings are defined per the 2000 Dietary Guidelines for Americans as 1 cup of raw leafy vegetables or ½ cup of other cooked or raw vegetables

^b^final model co-variates: food security status, total energy in kcal, adult education, child age and child gender

^c^final model co-variates: food security status, adult education, child age and child gender

^d^final model co-variates: child age and child gender

^e.^ final model co-variates: child age and child gender

^f.^ final model co-variates: food security status, Total Energy in kcal, adult race, child age and child gender

^g.^ final model co-variates: food security status, child age and child gender

^h.^ final model co-variates: food security status, adult education, child age, adult race, child gender, household size

^i.^ final model co-variates: food security status, Total Energy in kcal, adult education, adult race, child gender, child race, household size, child age

### Vegetable variety and liking, home availability of vegetables, and BMI-z score

**Fig. 2:**
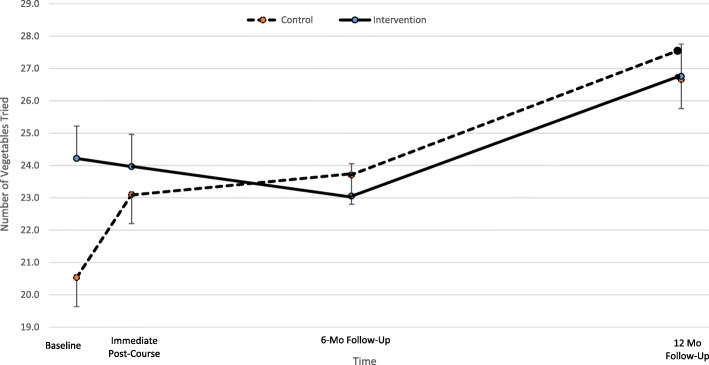
Within-group changes over time for mean number of vegetables (out of 37) tried at the four time points. Intervention parents were taught behavioral strategies to help increase their child’s vegetable intake. Error bars represent standard errors. Control:, Intervention

**Fig. 3:**
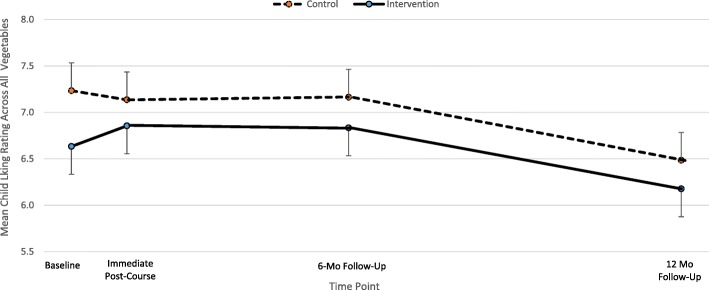
Within-group changes over time for mean child liking ratings across all vegetables. Children were asked to rate liking for 37 different vegetables. Ratings were made on a scale from 1 = “Hate it” to 10 = “Love it.” Intervention parents were taught behavioral strategies to help increase their child’s vegetable intake. Error bars represent standard errors. Control:, Intervention

Intervention children tried a larger variety of vegetables compared to control children only at baseline (*p* = 0.002). There were no other significant between-condition differences at any of the 4 time points for child vegetable liking and BMI-z score and home availability of vegetables (Table 4). Control children experienced a significantly larger increase in number of vegetables tried compared to intervention children from baseline to immediate post-treatment (LSM _control_ = 3.4, LSM _intervention_ = 0.05, *p* = 0.047) (Fig. 2). From immediate post-treatment to 12-month follow-up, both intervention and control children increased the number of total vegetables tried by 3 (intervention: 24 to 27, *p* = 0.02) (control: 23 to 26, *p* = 0.009) (Fig. 2). Although overall liking rating across all vegetables decreased for both conditions from baseline to 12-month follow-up, only the control children’s was significant (Fig. 3). No significant results were found for home availability of vegetables and child BMI-z score.

**Table 4:** Control vs. Intervention - Least Square Means of Child BMI-z, Liking, and Variety of Vegetables, and Home Availability of Vegetables at Baseline, Immediate Post-Treatment, 6 Mo Follow-up, and 12 Mo Follow-up

	Baseline			Immediate Post-Treatment			6 Mo Follow-Up			12 Mo Follow-up		
	Control	Intervention		Control	Intervention		Control	Intervention		Control	Intervention	
	LSM(SE)	LSM(SE)	t-test p-value	LSM(SE)	LSM(SE)	t-test p-val	LSM(SE)	LSM(SE)	t-test p-val	LSM(SE)	LSM(SE)	t-test p-value
Child BMIz score ^a^	0.9(0.2	0.8(0.2)	0.72	1.2(0.2)	0.8(0.2)	0.19	1.0(0.2)	0.8(0.2)	0.61	0.9(0.2)	0.8(0.2)	0.82
Mean Child Liking Rating of Vegetables^b,d^	7.2(0.3)	6.6(0.3)	0.20	7.0(0.3)	6.8(0.3)	0.71	7.0(0.3)	6.7(0.3)	0.51	6.4(0.3)	6.2(0.3)	0.65
Mean Number of Vegetables Tried^b,e^	20.3(0.9)	24.3(0.9)	**0.002**	23.0(1.0)	24.1(1.0)	0.42	23.4(1.0)	23.3(1.0)	0.92	26.3(0.9)	27.0(1.0)	0.63
Mean Number of Available Vegetables at Home^c^	16.2(1.3)	16.5(1.3)	0.85	17.4(1.4)	17.5(1.3)	0.96	16.1(1.4)	16.9(1.4)	0.67	15.9(1.3)	18.3(1.3)	0.22

a. final model co-variates: # days active 60 min, household size, and adult education

b. final model co-variates: # of available vegetables, adult education, child age, child gender

c. final model co-variates: food security status, adult education, household size, mean # of vegetables tried by adult, mean liking rating of vegetables by adult, child age, child gender

d. liking score range: 1 = “Hate it” to 10 = “Love it”

e. measure of variety

## Discussion

Overall, behavioral strategies that were explained and demonstrated to low-income parents within a parent-child cooking skills program and intended for implementation in their homes were generally not effective in improving the primary child outcome measures of this study. A number of possible factors may explain the lack of intervention effect. Parents may not have implemented the strategies in their homes as intended by the intervention. Parents were expected to take the strategies they learned about each week and practice them consistently at home. An assessment of the actual use of the strategies by parents relied on self-report. The self-reported habit index [46] was completed each week following the introduction of a new strategy and at all follow-up time points. Habit strength showed improvement immediately after the program for only 3 of the 6 strategies with declining strength over time (Additional file 2: Table S1), which may have contributed to the lack of intervention effect. The intention-behavior gap offers support towards non-practice of the strategies by parents in their homes [47]. During the strategy introduction segment of each class, participants expressed intention to practice the strategies. Once home, their intentions may not have translated into actually using the strategy because decisions regarding food choice are governed by more immediate reasons such as taste and convenience. Studies on similar strategies that have reported positive findings on vegetable choice and/or consumption have been set in school and other cafeteria environments allowing researchers more experimental control [19, 48]. Including a measure to capture evidence of strategy use could improve similarly delivered home interventions. The number of strategies, 6 in total, may have been too many for parents to implement as intended and practice enough to realize the hypothesized cumulative effects. In addition to implementing a new strategy each week, parents were encouraged to continue to use the previously introduced strategies. As such, parents were expected to use multiple strategies while serving vegetables with meals to their children, which may have become overly burdensome. Cravener and colleagues [26] focused on a single strategy in the home which may have encouraged more practice of the strategy, in turn, promoting actual behavior change. Moreover, tighter budget constraints of low-income households may make changes that would improve vegetable intake less of a priority or harder to execute [49]. For example, *Serve 2* and *Big Spoon* may have required the purchase of more vegetables than allowed by limited household budgets.

The results of this study are partly in line with recent work by Fulkerson et al. [11], that also enrolled parent-child pairs testing an intervention aimed to improve aspects of the home dietary environment including child vegetable intake. Both studies used a nutrition and cooking skills program format, delivered in a community setting with the expectation to implement what they learned in the program at home. Similar to the current study, Fulkerson and colleagues did not find an intervention effect on child vegetable intake, child BMI-z, and home availability of vegetables. These results considered together do not clarify if familial involvement in cooking meals at home would improve vegetable intake or associated outcomes.

Although there was no statistically significant intervention effect on the primary outcome measures, significant change-over-time measures for vegetable subgroups were observed. The intervention children’s significant increase in dark green vegetable intake from immediate post-treatment to 12-months suggests intervention parents may have increased inclusion of these types of vegetables into meals after completion of the cooking skills program. The following dark green vegetables were prepared throughout the cooking skills program: broccoli (sessions 5 and 6) collard greens (session 3), and spinach (sessions 2 and 6). The increase in dark green vegetable intake among intervention children may be promising considering this vegetable subgroup has been reported as one of the least consumed vegetable subgroups among this age group of children [40, 50]. One report estimated that only 10% of U.S. children aged 6–11 reported eating a dark green vegetable on a given day [51]. The significant difference in change-over-time measure (baseline to 6-months) where intervention children increased white potato intake and control children decreased intake is favorable given recent work touting the importance of white potatoes in a balanced diet [52, 53]. White potatoes are a significant source of high quality protein (i.e., digestibility and amino acid content), vitamins (ascorbic acid, B-6), minerals (potassium, magnesium, phosphorous), and phytochemicals [52]. The widely accepted perception that white potatoes are a *staple food* may have made it a popular choice in practicing certain strategies (e.g., Serve 2).

The increase in number of vegetables tried from baseline to 12-month follow-up for children in both treatment conditions is in the expected direction given the exposure to a variety of vegetables within the cooking skills program. Overcash and colleagues [54] used baseline and immediate post-treatment data from the combined intervention and control conditions of this study and found a significant increase for number of vegetables tried by the children. These findings are positive given the body of evidence indicating that increased exposure to a variety of vegetables is a precursor to increased liking and consumption of vegetables [35, 55].

The decline in overall vegetable liking for children in both treatment conditions may have prevented the hypothesized increase in vegetable intake and may be partially explained by the increase in number of vegetables tried. Participation in the nutrition and cooking skills program was expected to increase child liking of vegetables, due to increased exposure and the positive social experience paired with vegetables prepared and served in class, as posited by the evaluative conditioning theory [56]. The behavioral strategies were expected to increase child liking to an even greater extent. For example, the *Child Help* strategy exemplified the positive social interaction between parent and child in preparing vegetables for the meal, while *Serve 2* and *Big Spoon* strategies increased exposure to more vegetables. The increases in number of vegetables tried suggest the new vegetables tried by children may not have been liked [57] and therefore lowered the overall mean liking ratings.

A search of the relevant literature shows this may be the first large-scale controlled intervention trial set in the home that tested behavioral economics-informed strategies on increasing child vegetable intake. The racial and ethnic diversity of the study population, relatively low attrition, and use of a cooking skills program grounded in Social Cognitive Theory are the main strengths of the current study. The number of parent/child pairs that completed the assessments at immediate post-treatment (*n* = 91) was slightly lower than the targeted sample size of 100 and therefore may have been underpowered to detect differences between treatment conditions. The convenience sample of self-selecting participants may limit the generalizability of the findings. Lack of randomization of individual families into treatment conditions was also a limitation. Randomization of participant families was not possible given the low numbers of enrolled participant families at each location site. As such, each location site was designated a control or intervention treatment condition.

## Conclusions

Our findings suggest that the behavioral economics-informed strategies in this study and the manner in which they were implemented may not be effective in improving total vegetable intake for low-income children aged 9–12 years. However, the strategies may have potential for improving specific vegetable subtypes (e.g., dark green vegetables and white potatoes). The burden of implementing a number of strategies with potentially higher food costs may have constrained the ability of families in the current study to use the strategies as intended.

## Additional files

Additional file 1: **Figure S1**. Intervention Outcomes and Exposures based in Social Cognitive Theory [1]. (DOCX 33 kb)

Additional file 2: **Table S1**. Descriptive statistics for comparisons between four time points for the six behavioral strategies. (DOCX 21 kb)

Additional file 3: Raw data used in analysis. (XLSX 1624 kb)

## Data Availability

The dataset supporting the conclusions of this article is included as an Additional file 3 (file name: NCT03641521Datasets.xls).

## Abbreviations

BIC: Bayesian information criterion
*Big Spoon*: Behavioral strategy of using a bigger spoon to serve the vegetables
BMI: Body Mass Index
*Child Help*: Behavioral strategy of having your child help prepare vegetables for meals
HEI: Healthy Eating Index
LSM: Least square means
*Make Avail/Visible*: Behavioral strategy of making vegetables visible and accessible by removing other foods from the dining area during the meal and leaving the vegetables
*My Plat*e: Behavioral strategy of using a plate that shows the amount of vegetables to include for a meal
NCC: Nutrition Coordinating Center
NDSR: Nutrition Data System for Research
*Serve 2*: Behavioral strategy of serving at least 2 vegetables with the meal
*Serve First*: Behavioral strategy of serving vegetables before the meal
