# Supplementary material for: An in-home intervention of parent-implemented strategies to increase child vegetable intake: results from a non-randomized cluster-allocated community trial
Source: BMC Public Health. 2019 Jul 4;19:881. doi: 10.1186/s12889-019-7079-4 (PMC6610910; doi:10.1186/s12889-019-7079-4)
Supplement: Supplementary file 1 — Figure S1. Intervention Outcomes and Exposures based in Social Cognitive Theory [1]. (DOCX 33 kb) [file 12889_2019_7079_MOESM1_ESM.docx]

Additional file 1: Figure S1. Intervention Outcomes and Exposures based in Social Cognitive Theory[1]

Personal Factors

- Liking of Vegetables
- Self-Efficacy

Environmental/Social Factors

- Home availability of vegetables
- Parent & child cooking /learning together
- Parental role modeling
- Exposure to wide variety of vegetables

Behavior

- Vegetable intake
- Increased willingness to try new vegetables
- Child helping to prepare vegetables

1. Bandura, A. *Social foundations of thought and action : a social cognitive theory / Albert Bandura.*; 1986; ISBN 013815614X.
