# Supplementary material for: An in-home intervention of parent-implemented strategies to increase child vegetable intake: results from a non-randomized cluster-allocated community trial
Source: BMC Public Health. 2019 Jul 4;19:881. doi: 10.1186/s12889-019-7079-4 (PMC6610910; doi:10.1186/s12889-019-7079-4)
Supplement: Supplementary file 2 — Table S1. Descriptive statistics for comparisons between four time points for the six behavioral strategies. (DOCX 21 kb) [file 12889_2019_7079_MOESM2_ESM.docx]

**Additional file 2: Table S1. Descriptive statistics for comparisons between four time points for the six behavioral strategies.**

| Behavioral Strategy | Following Week  Mean (SD)^a^ | Immediately Post intervention  Mean (SD)^a^ | Immediately post-intervention (# of Weeks after Intro) | 6 month  Mean (SD)^a^ | 12 month  Mean (SD)^a^ |
| --- | --- | --- | --- | --- | --- |
|  |  |  |  |  |  |
| Child Help | 2.6 (0.8) | 2.9 (0.7)^b^ | 6 | 2.8 (0.8) | 2.8 (0.7) |
| MyPlate | 3.1 (0.7) | 3.1 (0.7) | 5 | 2.9 (0.8) | 2.6 (0.8) |
| Make Avail/Visible | 2.6 (0.8) | 2.8 (0.7)^b^ | 4 | 2.7 (0.7) | 2.5 (0.9) |
| Serve Two | 2.8 (0.7) | 3.1 (0.6)^b^ | 3 | 2.7 (0.8)^c^ | 2.9 (0.7) |
| Serve First | 2.9 (0.7) | 2.9 (0.7) | 2 | 2.7 (0.8) | 2.6 (0.7) |
| Big Spoon |  | 3.1 (0.6) | 1 | 2.9 (0.7) | 3.0 (0.7) |

^a^ T1 n varied between 37-42 by strategy; T2 n varied between 42-44 by strategy; T3 n varied between 25-29 by strategy; T4 n varied between 16-17 by strategy.

^b^ Significant difference between T1 and T2 according to paired T-test where the significance level is set at p< 0.017. (Bonferroni correction applied with three tests per strategy (0.05/3 = 0.017)

^c^ Significant difference between T2 and T3 according to paired T-test where the significance level is set at p< 0.017.
